# Population and coherence dynamics in large conjugated porphyrin nanorings†

**DOI:** 10.1039/d2sc01971j

**Published:** 2022-07-26

**Authors:** Giovanni Bressan, Michael Jirasek, Palas Roy, Harry L. Anderson, Stephen R. Meech, Ismael A. Heisler

**Affiliations:** School of Chemistry Norwich Research Park, University of East Anglia Norwich NR4 7TJ UK s.meech@uea.ac.uk; Department of Chemistry, University of Oxford, Chemistry Research Laboratory Oxford OX1 3TA UK; Instituto de Física, Universidade Federal do Rio Grande do Sul Avenida Bento Gonçalves, 9500, CEP 91501-970 Porto Alegre Brazil ismael.heisler@ufrgs.br

## Abstract

In photosynthesis, nature exploits the distinctive electronic properties of chromophores arranged in supramolecular rings for efficient light harvesting. Among synthetic supramolecular cyclic structures, porphyrin nanorings have attracted considerable attention as they have a resemblance to naturally occurring light-harvesting structures but offer the ability to control ring size and the level of disorder. Here, broadband femtosecond transient absorption spectroscopy, with pump pulses in resonance with either the high or the low energy sides of the inhomogeneously broadened absorption spectrum, is used to study the population dynamics and ground and excited state vibrational coherence in large porphyrin nanorings. A series of fully conjugated, alkyne bridged, nanorings constituted of between ten and forty porphyrin units is studied. Pump-wavelength dependent fast spectral evolution is found. A fast rise or decay of the stimulated emission is found when large porphyrin nanorings are excited on, respectively, the high or low energy side of the absorption spectrum. Such dynamics are consistent with the hypothesis of a variation in transition dipole moment across the inhomogeneously broadened ground state ensemble. The observed dynamics indicate the interplay of nanoring conformation and oscillator strength. Oscillatory dynamics on the sub-ps time domain are observed in both pumping conditions. A combined analysis of the excitation wavelength-dependent transient spectra along with the amplitude and phase evolution of the oscillations allows assignment to vibrational wavepackets evolving on either ground or excited states electronic potential energy surfaces. Even though porphyrin nanorings support highly delocalized electronic wavefunctions, with coherence length spanning tens of chromophores, the measured vibrational coherences remain localised on the monomers. The main contributions to the beatings are assigned to two vibrational modes localised on the porphyrin cores: a Zn–N stretching mode and a skeletal methinic/pyrrolic C–C stretching and in-plane bending mode.

## Abstract 2

Pump wavelength-dependent, ultrafast excited state dynamics arising from inhomogeneous broadening and ground and excited state nuclear wavepackets were observed for a series of Zn porphyrin nanorings made of 10 to 40 repeating units.

## Graphical abstract

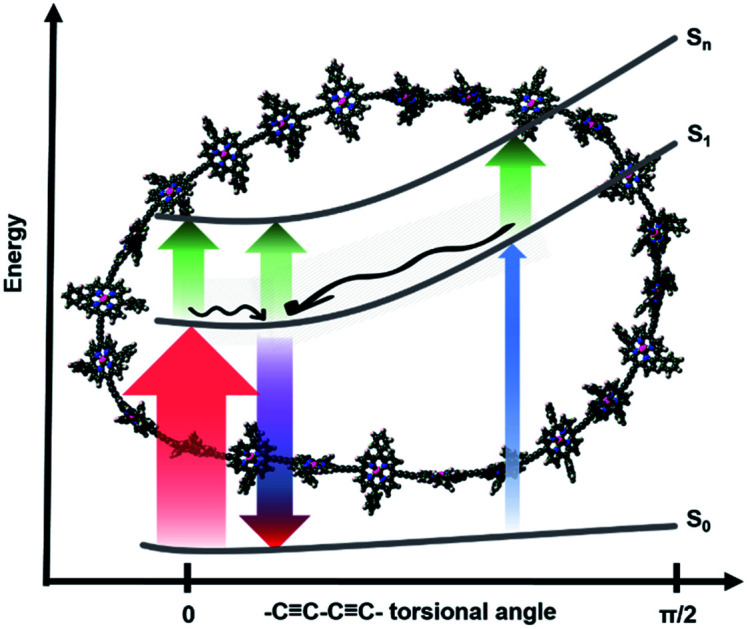

## Introduction

Tetrapyrroles have been extensively employed as building blocks of supramolecular biomimetic structures and as model systems for energy transfer between coupled chromophores.^1,2^ Their photophysics have been investigated with both steady-state and ultrafast time-resolved spectroscopic techniques.^3–8^ Butadiyne-linked zinc porphyrin nanorings are synthetic conjugated supramolecular nanosystems with distinctive topologies, such as cyclic (nested) rigid or flexible structures,^9^ and have a rich energy level structure, which has been rationalised in terms of Hückel theory.^10–12^ Their electronic excitations display highly nonlocal character^13–16^ giving them a resemblance to naturally occurring, bacteriochlorophyll-based, systems such as B800 and B850 in LH2,^17–19^ and as such represent important model systems for the study of exciton dynamics.

Among the key characteristics of natural light harvesting supramolecular chromophore assemblies, two are addressed in this study of synthetic nanorings. First is the role of static disorder, which shifts chromophore site energies, thus funnelling the electronic excitation energy toward the reaction centre.^18,20–22^ In this work, we provide a detailed picture of structural dynamics and disorder in porphyrin nanorings, and describe how it relates to more efficient light harvesting. Second is the nontrivial coupling between electronic and nuclear degrees of freedom,^23–26^ possibly boosting excitation energy transfer efficiency^23,27–29^ and giving rise to “quantum beats” in sub-ps domain transient spectra due to coherent nuclear motion in the ground and excited electronic states.^30,31^ In this work we identify and assign the origin of the dominant vibrational coherences in porphyrin nanorings, and discuss how they might contribute to enhanced energy or electron transfer in – for example – photovoltaic devices.

In studies of “quantum beating” phenomena it can be difficult to separate the features of ground and excited state vibrational wavepackets observed in fs transient absorption (fsTA). This is especially true in systems with small Huang–Rhys factors, such as chlorophylls,^32^ where partial overlap of the ground state bleach (GSB) and stimulated emission (SE) occurs, intertwining signatures of ground and excited state vibrational coherences.^33,34^ This picture can be further complicated by nonresonant contributions from solvent Raman active modes.^31,35^ Multidimensional spectroscopic techniques (in which information is spread over two, or more, frequency dimensions)^33,36–39^ provide a rigorous means of discriminating between ground and excited state vibrational coherences, but at the price of a more challenging experimental implementation and longer acquisition times. A feature which helps to differentiate between ground and excited state vibrations in conventional (one-dimensional) fs transient spectroscopies is the amplitude dip (or “node”) appearing at probe wavelengths corresponding to the maxima of the steady-state electronic absorption (for ground state nuclear wavepackets) or fluorescence (for excited state nuclear wavepackets) spectra. At these spectral positions, an abrupt π phase jump (“phase-flip”) of the oscillations occurs. Out-of-phase interference causes the amplitude of the oscillations to cancel at these wavelengths. Such a phenomenon has been previously described in the framework of a wavepacket evolving over time on a potential energy surface within a displaced harmonic oscillator model.^33,37,40–42^

The impact of static disorder (inhomogeneous broadening) and coherently excited nuclear motion on exciton dynamics has been extensively investigated and modelled in the natural light-harvesting complexes.^43–47^ Less attention has been dedicated to investigating how such factors affect the photophysical behaviour of their man-made counterparts.^19,48–50^ Here we investigate the fs-to-ns population and sub-ps coherent dynamics of porphyrin nanorings, with size ranging from 10 to 40 chromophore units, by means of visible fs transient absorption with tuneable broadband excitation. The central wavelength of the pump pulses is tuned to be in resonance with either the blue or the red edges of the nanoring's steady-state absorption spectra. This reveals an excitation energy-dependent spectral profile and (coherent) dynamical behaviour in porphyrin nanorings. Such effects have previously been observed in inhomogeneously broadened ensembles such as linear conjugated oligomers and polymers^34,51^ and bacteriochlorophyll light-harvesting ring structures, but were not previously studied in size controlled conjugated nanorings.^52,53^

## Results and discussion

### Steady-state electronic absorption and photoluminescence

**Fig. 1:**
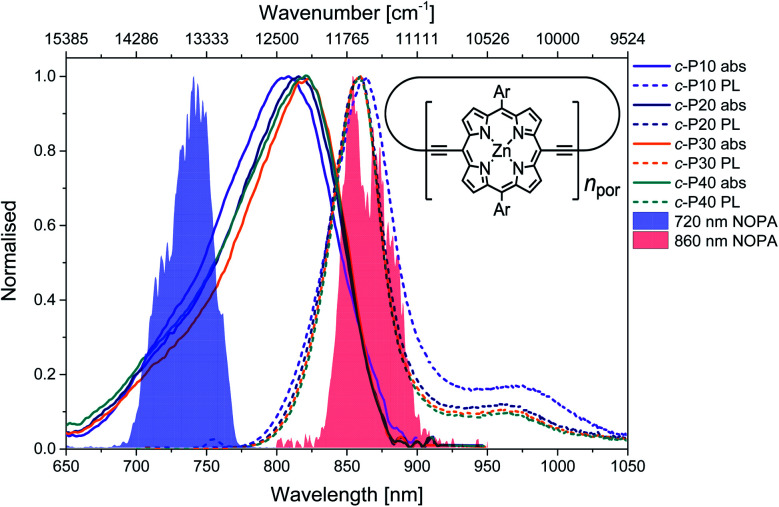
Normalised steady-state absorption (solid lines) and photoluminescence (dashed lines, excitation at 750 nm) of *c*-P10 (violet) *c*-P20 (navy), *c*-P30 (orange) and *c*-P40 (teal). The normalised pump pulse spectra are shown as blue (720 nm) and red (860 nm) shaded areas. A sketch of the molecular structure of the cyclic conjugated porphyrin nanorings is shown in the inset. Ar = 3,5-bis(octyloxy)phenyl.

The structure of the cyclic conjugated porphyrin nanorings is shown in the inset of Fig. 1. (Ar = 3,5-bis(octyloxy)phenyl). Normalised absorption and photoluminescence (PL) spectra are shown in Fig. 1. Steady-state electronic absorption and PL data in toluene with 1% pyridine have been previously reported and discussed,^11,13^ and are reproduced here to allow comparison to the noncollinear optical parametric amplifier (NOPA) pump pulses centred at either 720 nm (shaded blue area) or 860 nm (shaded red area).

Briefly, in large conjugated porphyrin nanorings, the HOMO–LUMO energy gap is inversely proportional to the number of repeating units (*n*_por_, chromophores) over which the excitation is delocalised. This explains the redshift of the absorption spectra accompanying the increase in ring size from *c*-P10 to *c*-P30. No redshift is observed between *c*-P30 and *c*-P40 because the delocalisation length saturates at around 20 porphyrin units.^11,13^ The spectral bandwidth of large nanorings is a trade-off between exchange narrowing, yielding a narrower bandwidth for longer conjugation lengths, and line broadening effects arising from structural disorder, which is greater for larger rings. The combination of these two effects produces the observed narrowing from *c*-P10 to *c*-P30 followed by a slight broadening in the spectrum of *c*-P40.^11^

The asymmetrical and broad spectra (Fig. 1) suggest static disorder in the ground state of large porphyrin nanorings, arising from a wide distribution of torsional angles between neighbouring porphyrins.^10,54^ As the angle between adjacent porphyrins affects both the exciton delocalisation length and the transition dipole moment,^54^ the oscillator strength of different subsets of the broadened ground state population can differ significantly. At every wavelength, the optical density is proportional to both the average number of molecules 〈*n*〉 and the square modulus of the transition dipole moment |*μ*|^2^. Therefore, we can suggest that the high-energy side of the steady-state absorption spectrum arises from nanorings with a more “twisted” conformation along the butadiyne axis. These will display a weak transition dipole moment and shorter conjugation length. On the other hand, the low-energy side of the absorption spectrum is assigned to a population of nanorings whose torsional angles are small, thus allowing for more extended delocalisation length and stronger transition dipole moments. This assignment is in agreement with a previous experimental and theoretical study of a butadiyne linked Zn porphyrin dimer.^55^

Normalised photoluminescence spectra of *c*-P*n*_por_ are shown as dashed lines in Fig. 1. The emission spectra of *c*-P20–40 are substantially overlapping, peaking at 860 nm, consistent with the similar nature of the excited states of *c*-P20–40, while the emission maximum of *c*-P10 is shifted to slightly lower energy (865 nm). The bandwidth of the photoluminescence spectra are narrower than the absorption spectra. This suggests that the emission comes from a narrower distribution of nanoring structures following excitation by (relatively) narrowband 750 nm excitation pulses (100 fs, 9 nm FWHM),^13^ suggesting some excited state evolution. Furthermore, photoluminescence spectra have a peak at 970 nm (975 nm for *c*-P10). This feature is most likely vibronic in origin.

Molar extinction coefficients and photoluminescence quantum yields of *c*-P10–40 are summarised in Table 1. In terms of absolute numbers, as expected, the molar extinction coefficients scale linearly with the number of chromophores per nanoring. The photoluminescence quantum yields for *c*-P20–40 are similar (within the uncertainty), in agreement with the exciton delocalisation length displaying a saturation behaviour for ring sizes above 20 porphyrin units. The reduced PL quantum yield of *c*-P10 is consistent with its electronic structure being intermediate between the larger nanorings and smaller rings with fully delocalised belt-like structures, which have much lower PL quantum yields.^12^ These data were previously reported by Parkinson *et al.*^13^

**Table 1:** Molar extinction coefficients and photoluminescence quantum yields for *c*-P10–40

Sample	Molar extinction coefficient [M^−1^ cm^−1^]	Photoluminescence quantum yield [% ±2.5%]
*c*-P10	630 650	11.4
*c*-P20	1 261 301	17.6
*c*-P30	1 891 952	19.2
*c*-P40	2 522 602	17.2

### Population dynamics: fs visible transient absorption

**Fig. 2:**
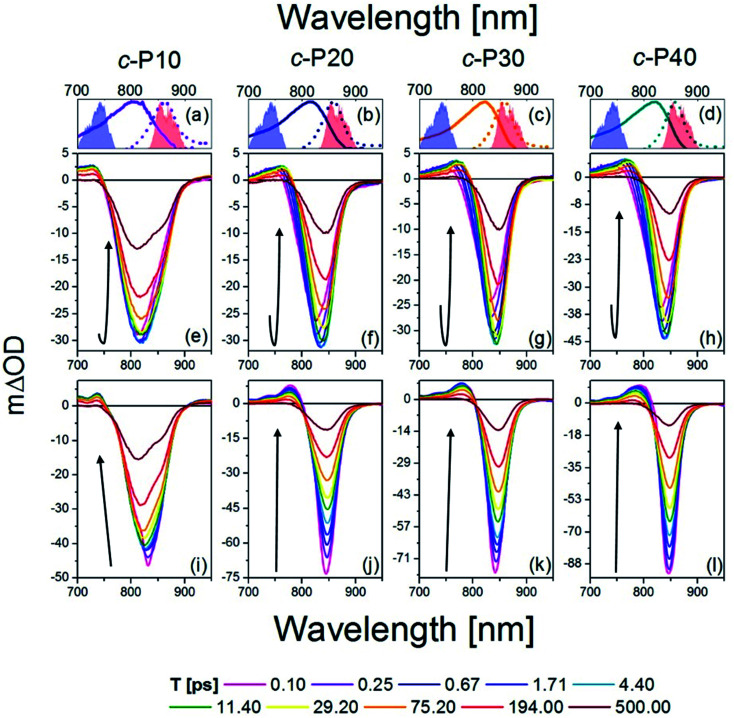
fsTA data between 0.1 ps and 500 ps after excitation of *c*-P10 (e, i), *c*-P20 (f, j), *c*-P30 (g, k), and *c*-P40 (h, l) with 720 nm (e–h) or 860 nm (i–l) centred pump pulses. The pump spectra are shown as blue (720 nm) or red (860 nm) shaded areas in the top panels. Top panels (a–d) also report the steady state absorption and photoluminescence spectra of each nanoring as solid and dashed lines, respectively, following the colour code used in Fig. 1.

The transient absorption spectra of *c*-P*n*_por_ at ten, logarithmically spaced, pump-probe delay times (*T*) between 0.1 and 500 ps are shown in Fig. 2e–h for excitation with 720 nm centred pump pulses and in Fig. 2i–l for excitation with 860 nm centred pump pulses. The energy was adjusted to 10 nJ for both 720 and 860 nm excitation pulses. This low energy limit was determined by Bressan *et al.* in their study of exciton–exciton annihilation in large porphyrin nanorings.^11^ To aid assignment, steady-state absorption (solid lines) and photoluminescence (dashed lines) of *c*-P*n*_por_, together with the spectra of the NOPA pulses (blue and red shaded areas) used for the two series of measurements, are shown in panels a–d.

The main feature of the transient absorption spectra of *c*-P*n*_por_ after 720 nm excitation is a prominent negative band, present from the earliest times (as shown for *T* = 0.1 ps, solid magenta lines) with minima at 811 nm for *c*-P10, at 825 nm for *c*-P20, and at 830 nm for *c*-P30 and *c*-P40. These signals are slightly red-shifted with respect to the maxima of the steady-state absorption spectra of each nanoring. This red-shift reflects a contribution from prompt SE from the Franck–Condon state. Negative bands are thus assigned to a convolution of the bleaching of the ground state population (GSB) and stimulated emission (SE). Unusually, these negative bands show an increase in amplitude (*i.e.* a deeper negative signal) as well as a redshift as a function of delay, *T*. This results in a noticeable band reshaping and redshift over the first 10 ps after photoexcitation, and the minima of the transient spectra at *T* = 10 ps appear at 820 nm for *c*-P10, and 845 nm for *c*-P20–40. The absolute amplitude growth of the region between 825 and 880 nm of the negative band is small for *c*-P10 but becomes more prominent as the size of the rings increases. The region of negative signal spectrally matches the wavelengths for which *c*-P*n*_por_ photoluminescence is detected and is thus assigned to stimulated emission (SE) from a region of the excited state potential energy surface which is not promptly populated by 720 nm excitation. On longer timescales (10 to 100 ps), the negative minima of *c*-P*n*_por_ are further shifting to the red in *c*-P20–40. The positive signals between 730 and 780 nm on the blue side of the negative bands are assigned to S_1_ → S_*n*_ excited state absorption (ESA) contributions, whose risetime and redshift in the first 10 ps followed by slower, further shift to the red and relaxation to baseline in 500 ps in *c*-P20–40, match the dynamical evolution of the negative (GSB + SE) bands. A broad, weak and featureless ESA between 1000 and 1200 nm is present from the earliest measured pump-probe delay time. Its dynamics are remarkably different from the 760 nm ESA, as this NIR ESA displays non-monoexponential relaxation to baseline within 1 ns without any evidence of a risetime (see traces in Fig. S2†).^11^

The dynamics retrieved upon excitation by 860 nm centred pump pulses (red shaded areas in Fig. 2a–d) are strikingly different, as neither a fast increase in amplitude of the negative band, nor a redshift are observed. The earliest time transient spectra (*T* = 0.1 ps) show an intense narrow negative feature peaked at 825 nm for *c*-P10 and 842 nm for *c*-P20–40. In sharp contrast to 720 nm excitation, these signals exhibit a 20% amplitude decrease within the first 1–2 ps, followed by slow redshift and non-monoexponential decay over tens to hundreds of ps. In *c*-P10 only, asymmetrical broadening towards the blue occurs over the same timescale, giving rise to a double minimum band, as observed (over the same timescale) in the data obtained for 720 nm excitation. Such features, not observed in the transient spectra of larger porphyrin nanorings, resemble the spectral features of smaller, fully delocalised *c*-P6, *c*-P6T6 and *c*-P8,^10,54^ and therefore can be assigned to the fully delocalised nature of the electronic states of *c*-P10. The positive ESA signals, between 740 and 790 nm have maximum amplitude at *T* = 0.1 ps and experience multiexponential relaxation to baseline within 500 ps, without any significant spectral shifts. In both pumping conditions, the negative bands of *c*-P*n*_por_ are not completely recovered even at the longest experimentally measured pump-probe delay time. The NIR ESA dynamics closely match those observed with 720 nm pump.

To highlight the dissimilar kinetics of the negative (GSB + SE) band in *c*-P*n*_por_ after excitation at different wavelengths, a comparison of the time traces (probe wavelengths = 842 nm and 1100 nm) of *c*-P40 excited at 720 and 860 nm is reported, as an example, in Fig. S2.† A comparison of the transient spectra of *c*-P40 excited at 720 or 860 nm for *T* = 0.1, 4.5 and 500 ps is shown in Fig. S3.† Similar excitation wavelength dependent dynamics and spectral shapes were reported by Chang *et al.*^54^ in a time-resolved photoluminescence study of a linear, butadiyne-linked, porphyrin octamer.

Despite using the same pump pulse energy (10 nJ) at both the 720 and 860 nm, and the convolution between the normalised steady-state absorption and pump spectra being 15% larger for the 720 nm pump, the amplitude of the negative (GSB + SE) signals is much smaller when *c*-P*n*_por_ are excited at 720 nm (*m*ΔOD = −30, −45 at *T* = 5 ps) than at 860 nm (*m*ΔOD = −50, −90 at *T* = 0.1 ps) (Fig. 2e–l). Such a difference is in agreement with the assignment of transition dipole moment strength increasing toward the red side of the inhomogeneously broadened ground state nanoring population. The possibility of the difference in amplitude being due to a larger excited state population created by the 860 nm excitation is further ruled out by the behaviour of the broad ESA between 1050–1200 nm (ESA traces at 1100 nm shown in Fig. S2†). This signal does not show any appreciable amplitude variation as a function of the excitation wavelength (being specifically 6 *m*ΔOD for 720 nm pump, 5 *m*ΔOD for 860 nm pump at *T* = 0.1 ps).

**Fig. 3:**
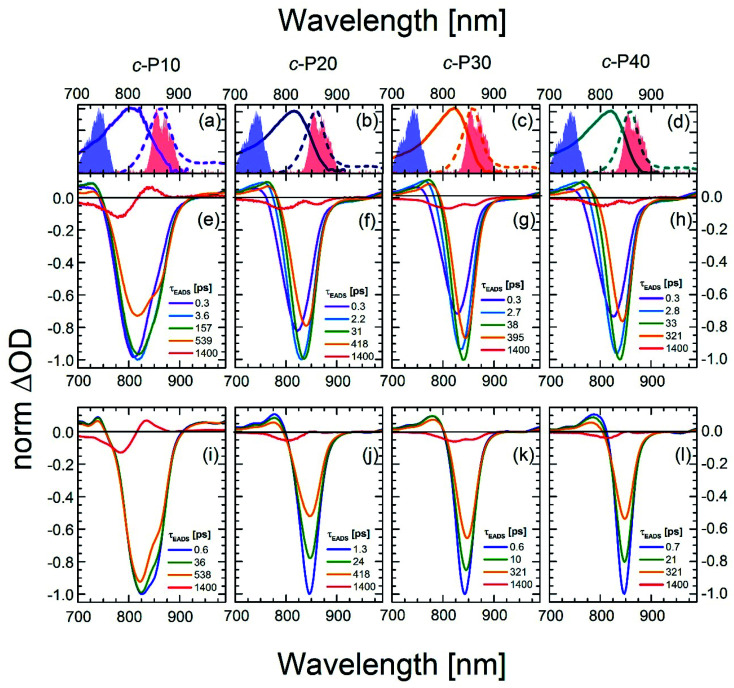
Normalised evolution associated difference spectra (EADS) obtained by global fitting of the fsTA data of *c*-P10 (e, i), *c*-P20 (f, j), *c*-P30 (g, k), and *c*-P40 (h, l) after excitation with 720 nm (e–h) or 860 nm (i–l) centred pump pulses. The pump spectra are shown as blue (720 nm) or red (860 nm) shaded areas in the top panels. Top panels (a–d) also report the steady state absorption and photoluminescence spectra of each nanoring as solid and dashed lines, respectively, following the colour code used in Fig. 1.

To quantify the dynamical evolution of the fs transient absorption spectra, and highlight the different dynamical behaviour observed by tuning the pump wavelength, a global fitting analysis (using Glotaran)^56^ was applied to all the datasets, assuming sequential evolution through a series of first-order steps. The resulting normalised evolution-associated difference spectra (EADS) are reported in Fig. 3. Due to the similarities in their transient photophysical behaviour, the EADS of *c*-P20–40 are discussed together.

**Fig. 4:**
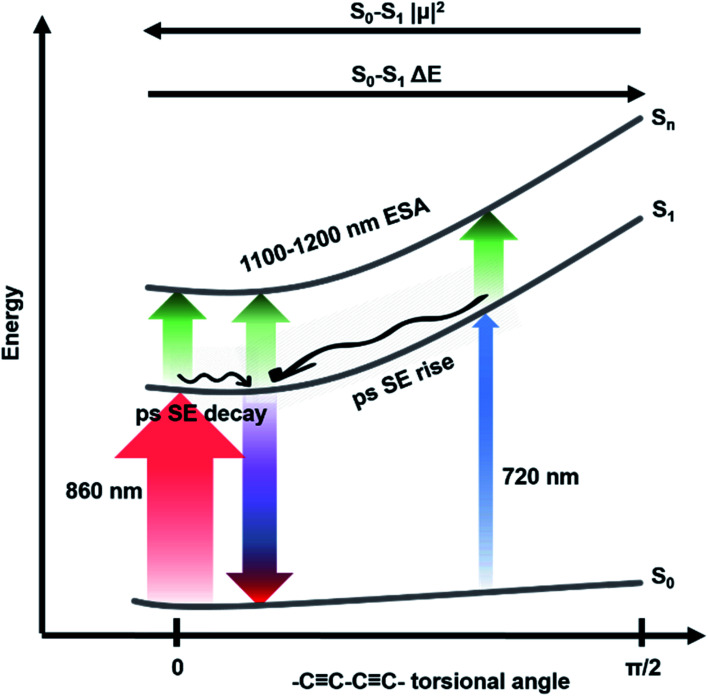
Level diagram representing ground and excited states and fast dynamics of *c*-P*n*_por_. Transitions observed *via* fsTA after excitation with ultrashort 720 nm (blue upward arrow) or 860 nm (red upward arrow) pump pulses. Stimulated emission from the minimum of the excited state potential energy surface is represented as a blue/red downward arrow. S_1_ → S_*n*_ NIR excited state absorption is represented as green upward arrows. The arrows thickness is proportional to the transition dipole moment of each transition. Relaxations in the excited state potential energy surface are represented by wavy arrows. Assignments are reported as well.

Five components were necessary to obtain good fits to the experimental data of *c*-P*n*_por_ excited at 720 nm. The two fastest components (0.3 ps, violet solid lines in Fig. 3e–h and in the 2.2–2.8 ps range in *c*-P20–40, light blue solid lines in Fig. 3f–h) account for the redshift and a 20–25% relative amplitude increase of the negative band. We assign this red-shifting and strengthening (deeper) bleach signal to the development of a more intense stimulated emission, as a function of time after photoexcitation on the blue side of the steady-state absorption. The positive excited state absorption signals around 750 nm display the same red-shifting and rising behaviours, which are not reflected in the EADS region between 1050 and 1200 nm. Such SE and ESA rise times and redshifts may arise from non-single exponential relaxation of the initial exciton toward a region of the excited state potential energy surface (PES) with increased transition dipole moment along an inhomogeneously broadened distribution of structures (this is illustrated in Fig. 4). The non-single exponential nature of the relaxation dynamics may suggest more than one coordinate is involved in the excited state structural relaxation. Similar phenomena have been observed for singlet excitations of highly conjugated organic oligomers and polymers.^57,58^ Additional evidence for inhomogeneity in *c*-P*n*_por_ is given by the linewidth of the emission spectra (dashed lines in panels a–d of Fig. 3) obtained upon narrowband excitation (∼9 nm FWHM) at 750 nm.^13^ The emission spectra are substantially narrower than the corresponding absorption features (solid lines in panels a–d of Fig. 3).

A smaller red-shift and amplitude increase of the negative band is observed on a comparable timescale in *c*-P10. *c*-P10 is in the transition regime between the fully delocalised nanoring *c*-P6, which does not show a delayed formation of the SE band on blue edge excitation,^10^ and the larger *c*-P20–40 rings, whose exciton coherence lengths do not span the whole ring circumference.

The EADS formed in *ca.* 3 ps (dark green solid lines) relax in the range of 31–38 ps in *c*-P20–40 yielding a further redshift of both the SE and the 760 nm centred ESA band. The risetime and redshift of this 760 nm ESA match the evolution of the (GSB + SE) negative signal. The same EADS also reveals a 20% amplitude reduction to yield the orange EADS, which itself relaxes in hundreds of ps. The 31–38 ps relaxation times are assigned to further slower structural dynamics involving the planarisation of the dihedral angle between neighbouring porphyrin units, but now coupled with the onset of population relaxation leading to bleach filling. Analogous dynamics were observed, on a slower timescale (200 ps), for a six-membered porphyrin ring of the same family.^10,59^ We speculate that this slower planarisation is a function of ring circumference, as larger rings present more flexible structures and reduced strain. This is consistent with the planarization in *c*-P10 excited at 720 nm (solid dark green line in Fig. 3e) which has a time constant of *ca.* 160 ps, intermediate between the values measured for *c*-P6 and *c*-P20–40. The second longest component (solid orange lines in Fig. 3e–h) with time constants in the region of 300 to 600 ps, account for an overall decay of the relaxed transient spectra due to population relaxation back to the ground state. The minor long-lived components (solid red lines in Fig. 3e–h) are assigned to the incomplete recovery of the ground state due to some population being trapped in a long lived triplet state (whose lifetime cannot be experimentally resolved by our experiment) as a consequence of intersystem crossing (ISC). Similar behaviour was observed with fsTA and cryogenic temperature transient EPR spectroscopies, in smaller porphyrin nanorings.^10,60^

The data obtained upon excitation of *c*-P*n*_por_ with 860 nm centred pump pulses required four time components to be properly fit, one less than in the 720 nm pump case. The fastest (0.6–1.3 ps range), and most intense EADS component (solid dark blue lines in Fig. 3i–l) are narrower and red-shifted compared to the earliest EADS obtained when the same molecules are excited by 720 nm centred pump pulses, reflecting the inhomogeneity in the ground state population. In fact, the fastest evolution-associated difference spectra measured after 860 nm excitation match the EADSs observed a few ps after 720 nm pumping (solid dark green lines in Fig. 3e–h). This suggests that, when *c*-P*n*_por_ are excited with pump pulses resonant with the red edge of their absorption spectrum, the low-energy, strongly emissive, sites within the inhomogeneous distribution are directly populated, giving rise to prompt, intense, stimulated emission signals centred at 850–854 nm.

The rapid amplitude decrease of this component is assigned to an energetically downhill relaxation toward the minimum of the excited state potential energy surface, with a transition dipole moment that is smaller compared to the, initially populated, Franck–Condon (FC) state. This is reflected in the *ca.* 20% decay of the SE amplitude on this timescale. Such a fast decay of the SE band could in principle indicate the presence of a rapid population decay back to the ground state on a ps timescale. However, the S_1_ → S_*n*_ excited state absorption (ESA) at 1050–1200 nm does not display any such fast relaxation, as shown by the time traces in Fig. S2† and in a previous work on exciton–exciton annihilation dynamics.^11^ We thus propose that the measured fast SE decay is due to relaxation of the, vertically excited, FC state to a region of the excited state PES with decreased transition dipole moment rather than to a population relaxation (Fig. 4).

The absence of a fast blue-shift accompanying the fast SE decay after 860 nm excitation can be explained through the dependence of S_0_ → S_1_ transition dipole moment (TDM) and transition energy on the torsional angle between neighbouring porphyrins.^10,55,59,61^ These have been measured and calculated for the Zn porphyrin dimer by Peeks *et al.*^55^ Their results show that the TDM has a linear dependence on the torsional angle, while the transition energy is a function of the cosine of the torsional angle. Thus, 860 nm centred pump pulses excite a population of rings whose torsional angle will be, on average, small, close to the value corresponding to the excited state PES minimum. Therefore, only a relatively small variation of the torsional angle is needed to reach the PES minimum in the excited state. Because at small angles the oscillator strength is more sensitive to the torsional angle than the transition energy (linear rather than cosine dependence), torsional relaxation on the excited state PES will give rise to the observed fast SE amplitude decay, but a negligible blue-shift. Note that this model suggests the excited state PES minimum corresponds to a small, but nonzero, torsional angle, as opposed to the ground state, as sketched in Fig. 4. We speculate that this difference arises from the more pronounced cumulenic nature of the S_1_ state compared to S_0_, in agreement with TD-DFT studies performed by Peeks *et al.*^55^

On longer timescales, the EADS of *c*-P*n*_por_ present a minimal red-shift of 3–4 nm and undergo non-monoexponential decay assigned to S_1_ population relaxation, with components in the tens and hundreds of ps range, closely resembling the dynamics observed upon 720 nm excitation over the same timescale, leading to nearly complete recovery of the ground state population on the ns timescale. The small residual, long-lived, GSB is again assigned to ISC. The weak positive signal centred at 833 nm in the longest EADS of *c*-P10, for both the 720 and 860 nm excitation conditions is therefore assigned to triplet–triplet (T_1_ → T_*n*_) excited state absorption.

Tuning the pump wavelength across the steady-state absorption profile of *c*-P*n*_por_ translates to remarkable differences in the early time transient spectra, consistent with an inhomogeneously broadened ensemble with a range of radiative transition moments and S_0_ → S_1_ energies as a function of the torsional angle.^52^ Similar observations were described by Peeks *et al.* for the butadiyne linked, Zn porphyrin dimer.^55^

To obtain a clearer picture of how such differences in the excited state dynamics affect the coherent coupling of vibrational modes to the electronic transitions, we analysed the femtosecond coherence spectra (FCS) of *c*-P*n*_por_.

### Coherent vibrational dynamics: fs coherence spectroscopy

Removing the relaxation dynamics by subtracting the multiexponential global fits (shown in Fig. 3) to the population dynamics from the fsTA data of *c*-P*n*_por_ (shown in Fig. 2) permits isolation of their sub-ps coherent behaviour, which arises from the generation and evolution of ground and excited state vibrational wavepackets.^37,40^

**Fig. 5:**
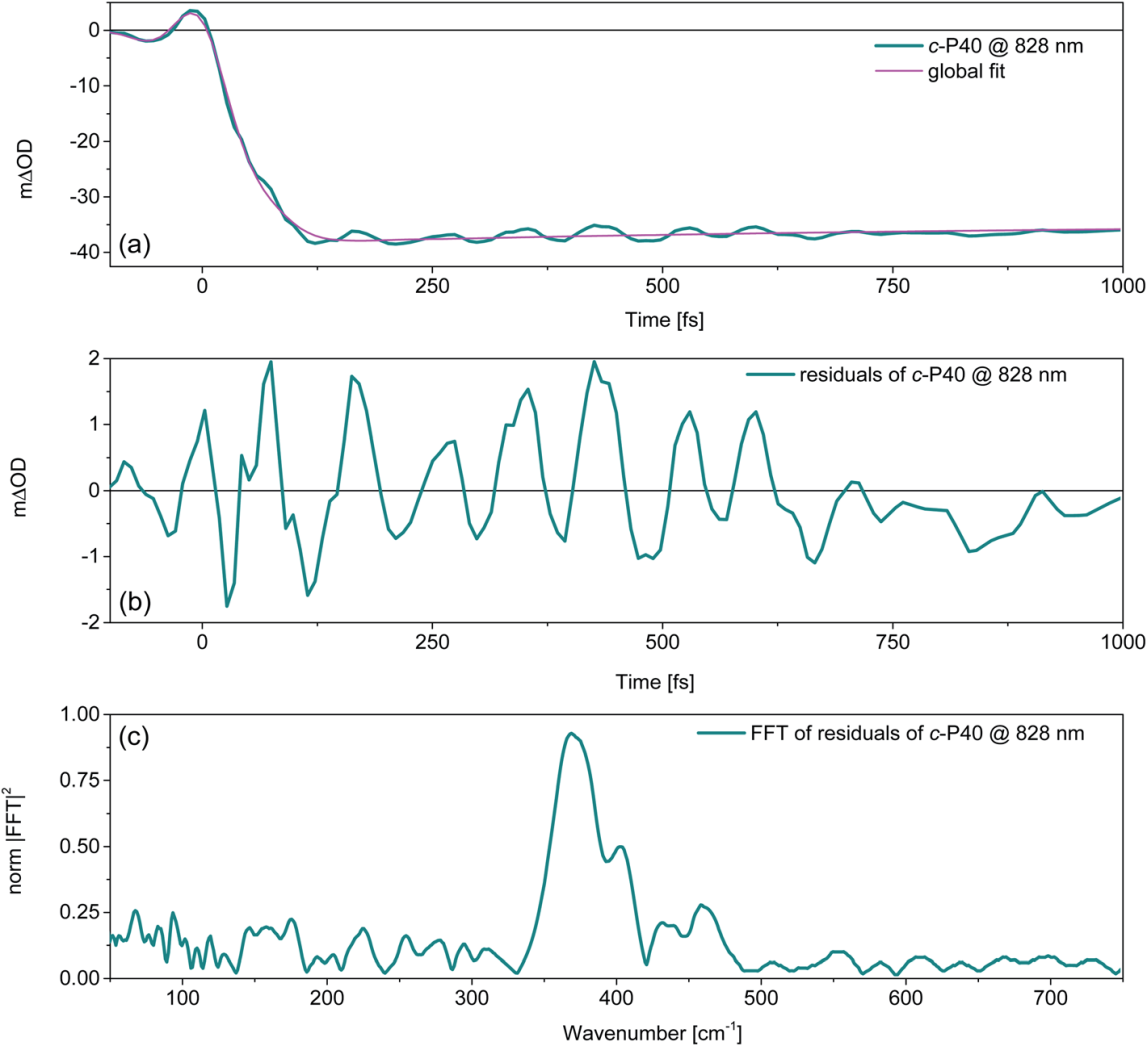
(a) Transient response of *c*-P40 (solid teal line) pumped with NOPA pulses centred at 860 nm, measured at a probe wavelength of 828 nm, showing fast oscillatory and slower relaxation dynamics. A trace at 828 nm taken from the global fit of the data is overlaid as a solid magenta line. The contribution of the sub-ps decaying component is minor at this wavelength as this trace was taken toward the blue edge of the negative (GSB + SE) signal, where oscillation amplitude is maximised. (b) Fast oscillatory behaviour of *c*-P40 isolated by the subtraction of the global fit from the experimental data. Note the difference between the maxima and minima of the ordinate axes of (a) and (b). (c) Fourier transform of the fast oscillations shown in (b), displaying a dominant resonance Raman mode at 370 cm^−1^.

**Fig. 6:**
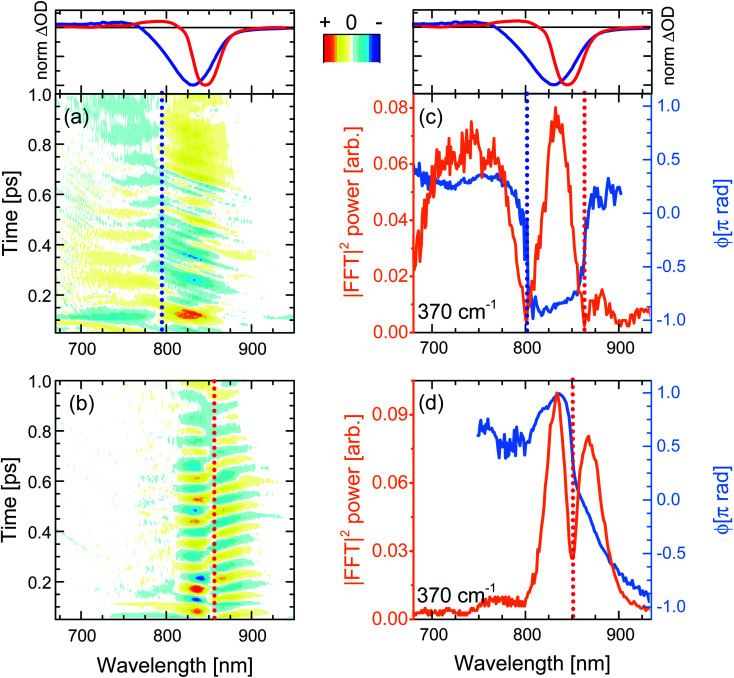
(a and b) Femtosecond coherence spectra of *c*-P40 after impulsive excitation by 720 or 860 nm broadband pump pulses. Numerical Fourier transformation of (a and b) yields phase and amplitude maps of the Raman active modes contributing to the time-domain beatings. (c and d) Probe wavelength-resolved amplitude (orange solid line) and phase (blue solid line) of the 370 cm^−1^ mode in the ground and excited electronic states of *c*-P40, respectively. Normalised fsTA spectra at *T* = 100 fs of *c*-P40 excited at 720 and 860 nm are shown in the top panels as blue and red solid lines as a reference. Spectral positions at which the amplitude nodes/phase-flips occur for ground and excited state coherences are highlighted by vertical dotted blue and red lines, respectively. Oscillatory amplitude in panels (a, b) is normalized to the maximum and has 21 evenly-spaced contour lines.

Fig. 5a shows an example of the behaviour of the transient oscillatory and slowly decaying response (for probe wavelength = 828 nm) obtained by pumping *c*-P40 with 860 nm centred NOPA pulses, overlaid with a trace from the global fit. The fast oscillatory response of *c*-P40, isolated by subtraction of the global fit is shown in Fig. 5b. The oscillatory behaviour has a *ca.* 85 fs period. The Fourier transform of this oscillation is shown in Fig. 5c and displays a dominant mode at 370 cm^−1^. Probe-wavelength resolved oscillatory behaviour is shown for *c*-P40 in Fig. 6a, b and for *c*-P10–30 in the ESI (Fig. S4†). A single damped sinusoid at 371 cm^−1^ with a dephasing time of 750 fs is sufficient to obtain a good fit to the oscillatory residuals. The extracted parameters are in good agreement with the Fourier domain data (Fig. S5b†).

When the pump pulses are centred at 720 nm, such beatings display an amplitude node (phase-flip) situated at 799 nm, which is on the blue side of both the steady-state absorption maximum (820 nm) and the fsTA GSB minimum (*T* = 100 fs, solid blue lines in top panels of Fig. 6), thus suggesting an assignment of these oscillations to a ground state vibrational coherence (*c*-P40 reported in Fig. 6a, *c*-P10–30 in Fig. S4d–f†). The presence of the amplitude node to the blue of the steady-state absorption maximum (which all *c*-P*n*_por_ display when excited on the blue side of their steady-state absorption spectra, see Fig. S4d–f†), is assigned to the inhomogeneous broadening (static disorder) in the ground state population of such large nanorings, as observed in the population dynamics discussed above. In the present case the 720 nm pump excites a blue shifted sub population. This observation indicates that the spectral diffusion in the ground state potential energy surface is slower than several picoseconds, such that the disorder experienced by the molecular ensembles is “frozen” over this timescale. The resulting broadening can thus be considered inhomogeneous on the timescales relevant for ground and excited state coherent nuclear wavepacket motion.

The static disorder permits selective excitation of a high-energy subset of nanorings within the inhomogeneous distribution by tuning the pump spectrum into resonance with the blue edge of the steady-state absorption spectra. Such a population experiences non-exponential fast motion toward a more allowed region of the excited state PES, with the fastest component relaxing with a time constant of 0.3 ps. We speculate that this fast relaxation promotes decoherence of the excited state vibrational wavepackets, as no oscillation with a node at a wavelength matching the photoluminescence maximum is observed in Fig. 6a. The time domain data only show a weak oscillation (600 *μ*ΔOD, Fig. S5a†) with a node at 799 nm due to the ground state vibrational wavepacket contributions.^62^ The small amplitude of the ground state coherent vibrational oscillations may arise from broadband pulses bleaching to the same extent a wide distribution of ground state nuclear vibrational coordinates (distinct from the, lower energy, torsional coordinate causing the inhomogeneity), thus hampering the generation of a net ground state moving “hole”.^37,62–65^

Numerical Fourier transformation of the time-domain data allowed retrieval of amplitude and phase of the 370 cm^−1^ mode as a function of the probe wavelength. These are shown as orange and light blue solid lines, respectively, in Fig. 6c. The presence of an abrupt π phase-jump at 860 nm (near the photoluminescence maximum) and of a weak peak between 860 and 900 nm suggest that, despite the absence of clear time-domain oscillations in this spectral region (Fig. 6a), residual excited state vibrational wavepackets are contributing to the coherent response of *c*-P40 excited by the 720 nm centred broadband pump pulses. The FT amplitude and phase profiles of *c*-P10–30 excited with NOPA pulses centred at 720 nm, on the blue side of their steady-state absorption spectra, show the same behaviour and are reported in Fig. S6.†

When *c*-P*n*_por_ are excited by pump pulses in resonance with the red side of their steady-state absorption, the resulting femtosecond coherence spectra (*c*-P40 reported in Fig. 6b, *c*-P10–30 in Fig. S4g–i†) display an oscillatory behaviour with *ca.* 85 fs period and a node at 852–854 nm, matching the minima of the intense negative features of the earliest fsTA spectra (100 fs, red lines in top panels of Fig. 6) and the photoluminescence maxima (dashed lines in Fig. 1). Such beatings modulate the amplitude of the SE as a function of the pump-probe delay time and are thus assigned to vibrational wavepackets evolving in a strongly allowed region of S_1_. In this case, the oscillatory part of the transient response is dominated by stronger (2–3 *m*ΔOD, Fig. S5a†) excited state vibrational coherence contributions, as there is no GSB amplitude (solid red lines in top panels of Fig. 6) between 750 and 800 nm, in the spectral region where the oscillatory features and node/phase-jump due to ground state vibrational wavepackets are expected to appear.

Fig. 6d contains the same information shown in Fig. 6c for the oscillations measured upon excitation with 860 nm pump pulses. Fig. 6d shows how the dip in the power spectra amplitude (orange line) is accompanied by a phase-jump (light blue line), appearing at a wavelength matching the photoluminescence maximum, in agreement with the time-domain data. Such an effect is also observed in the amplitude and phase profiles of *c*-P10–30 excited with NOPA pulses centred at 860 nm, on the red edge of their steady-state absorption (Fig. S6†).

Thus, the oscillatory features in the FCS of *c*-P*n*_por_ excited by ultrashort broadband pump pulses are explained in terms of generation and evolution of vibrational wavepackets on the ground and excited state potential energy surfaces. This assignment is based on the spectral position of the amplitude nodes at, or near, the maxima of steady-state absorption or photoluminescence, in the context of a large inhomogeneous broadening causing remarkably different excited state population evolution as a function of the excitation wavelength.

### Ground and excited state impulsive resonance Raman spectra

**Fig. 7:**
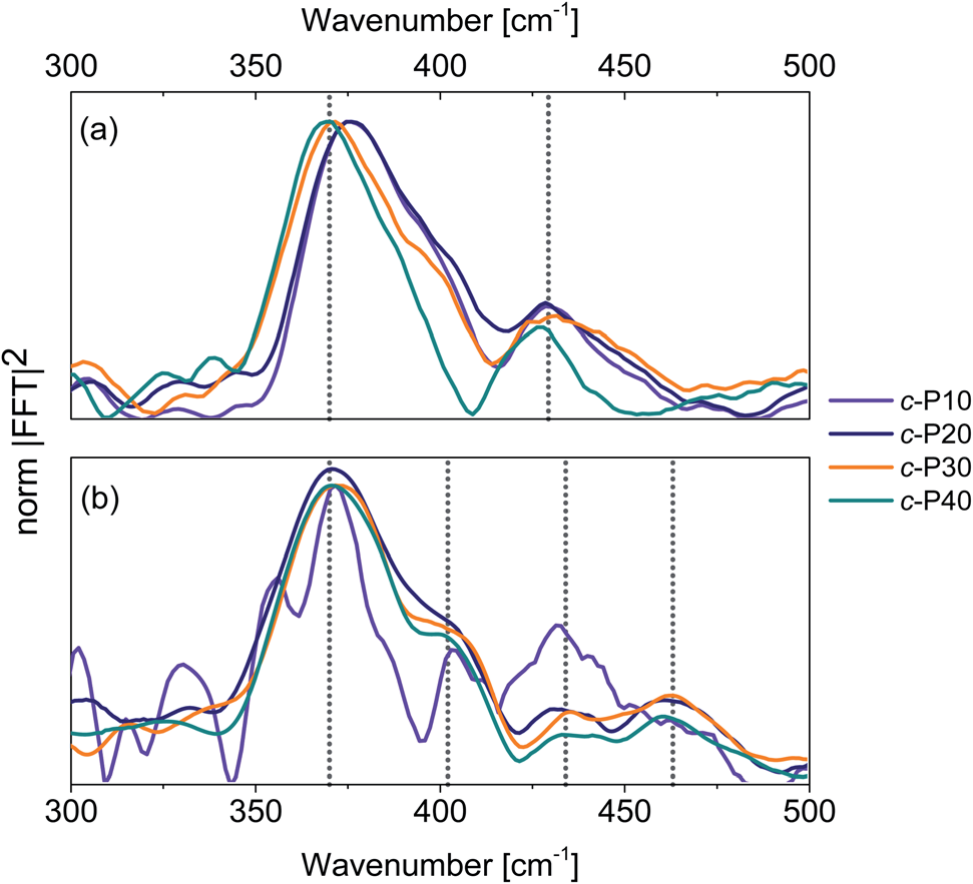
Impulsive Raman spectra of *c*-P10 (violet), *c*-P20 (navy), *c*-P30 (orange) and *c*-P40 (teal) of their ground (a) and excited (b) electronic states. These spectra are obtained by integration of the Raman maps shown in Fig. S6 and S7† over the probe wavelengths ranging from 720 to 850 nm (ground state Raman, a) or from 810 to 900 nm (excited state Raman, b) followed by normalisation at 370 cm^−1^. The signal-to-noise ratio of the ES impulsive resonance Raman spectrum of *c*-P10 (violet line in 7b) is low due to its small fsTA signal amplitude.

Integration of the probe wavelength-resolved Fourier transform of the time-domain oscillations shown in Fig. S7 and S8† over a limited range of probe wavelengths (700–800 nm for Raman maps obtained by 720 nm excitation, 810–900 nm for Raman maps obtained by 720 nm excitation) followed by amplitude normalisation yields the impulsive resonance Raman spectra of the ground and excited states of *c*-P*n*_por_ (Fig. 7a and b, respectively). An analogous procedure was adopted by Fitzpatrick *et al.* to obtain impulsive Raman spectra of the organic dye cresyl violet.^33^

The main contribution to the ground state Raman spectra of *c*-P30, 40 (Fig. 7a) is from the 370 cm^−1^ peak (blue-shifted by 5 cm^−1^ in *c*-P10, 20). Such a peak has been previously observed in the ZnTPP monomer,^66^ in which it is slightly blue-shifted, appearing at 380 cm^−1^. This dominant mode was detected in (templated) *c*-P6 at 380 cm^−1^ in a cryogenic temperature 2D electronic spectroscopy study of Butkus *et al.*^15^ This contribution is assigned to Zn–N bonds stretch in the porphyrin cores.^67^ The increase in ring size from *n*_por_ = 10 to 40 leads to a redshift, suggesting a reduction in the Zn–N force constant from the value in the monomer and six-membered ring. We speculate that the asymmetrical lineshape of these vibrational bands reflects the static disorder in the ground state ensemble.

A lower intensity mode peaking at 428 cm^−1^ is also observed. This signal can be assigned to a combination of stretching and in-plane bending of the pyrrolic and methinic C–C bonds, following the assignment of Atamian *et al.*^67^ for the parent ZnTPP monomer, in which the same mode is observed at 440 cm^−1^. Butkus *et al.* identified a similar mode at 440 cm^−1^ in (templated) *c*-P6.^15^ Our assignment is supported by DFT calculations on the Zn porphyrin monomer and butadiyne-linked dimer (cartoons of the Raman active ground state modes contributing to the vibrational coherences are shown in Fig. S9†). Such calculations were obtained in Gaussian 16 at the B3LYP/6-31G* level of theory. The close agreement between the coherent oscillations in the nanorings and the frequency observed in the monomer and in calculation suggests an assignment to a vibrational coherence.^68^ This is as opposed to vibronic coherences which arise from mixing between electronic and vibrational states, and may manifest as new frequencies.^68,69^ However, it is worth mentioning that a rigorous distinction requires two-dimensional electronic spectroscopy rather than one-dimensional transient absorption; such experiments are planned.

The excited state impulsive resonance Raman spectra of *c*-P20–40 (Fig. 7b) each display four signals, an intense peak at 370 cm^−1^ with a well resolved shoulder at 402 cm^−1^, and two weaker bands at 432 and 463 cm^−1^. The 370 cm^−1^ peaks were also observed in the corresponding GS impulsive Raman data and assigned to Zn–N stretching modes; these evidently do not display frequency shifts in the excited electronic states. This can be rationalised on the basis of the π* ← π nature of the underlying electronic transitions, whose charge density redistributions will only weakly influence molecular orbitals (MOs) involved in forming the Zn–N bonds.

We speculate that the shoulder centred at 402 cm^−1^ could arise from a redshift of the stretching and in-plane bending of the pyrrolic and methinic C–C bonds mode with B_1g_ symmetry, observed at 428 cm^−1^ in the GS resonance Raman. Zn porphyrin repeating unit of *c*-P*n*_por_ belong to the *D*_2h_ point group. In tetrapyrroles with *D*_2h_ symmetry, the B_1g_ MOs display bonding character between the pyrrolic carbon nuclei displaced by the (B_1g_) pyrrolic/methinic mode.^67,70^ Due to the bonding nature of the B_1g_ MO, the force constant of this vibrational mode is weakened in the S_1_ excited state,^70^ causing its redshift.

At higher wavenumbers, two weak peaks, centred at 433 and 462 cm^−1^, contribute to the ES resonance Raman spectra of *c*-P20–40. No assignment is proposed for the 433 cm^−1^ mode, while the peak at 462 cm^−1^ is not observed in the GS of *c*-P*n*_por_ and is assigned to a B_1g_ in-plane bending mode of the porphyrin backbone involving pyrrolic, methinic and phenylic (–Ar in the molecular structure shown in the inset of Fig. 2) nuclei.^67^ The calculated ground state frequency for this mode is 490 cm^−1^,^67^ but a redshift in the S_1_ state is expected, as discussed above for the pyrrolic/methinic B_1g_ mode. The appearance of this mode in the ES spectra only of *c*-P*n*_por_ is ascribed to a Raman scattering cross section enhancement of the methinic modes in the S_1_ state, as a result of hyperconjugation between the tetrapyrrole π system with the σπ MOs of the aryl side chains.^70^

It is interesting to note that the sub-ps coherent dynamics observed in *c*-P*n*_por_ are due to impulsive excitation of, ground and excited state, resonance Raman internal modes of the Zn porphyrin monomer. Such vibrations, localised on the monomeric units, are coupled to transitions between delocalised electronic states whose exciton size spans several chromophores. Similar conclusions were reached by Dean *et al.*^27^ who observed excited state vibrational coherences in light harvesting complexes, which were assigned to localised modes of constituent monomers. A recent polarisation-resolved 2D-ES study of the B850 complex also revealed that the vibrational modes coupled to its electronic transitions have monomeric (chlorophyll) character.^53^

## Conclusions

Population and coherent vibrational dynamics in fully-conjugated nanorings made up of 10 to 40, alkyne-bridged Zn–porphyrins were studied by femtosecond visible transient absorption spectroscopy using broadband pump pulses whose central wavelength was tuned to resonance with either the blue or red edges of the nanorings steady-state absorption spectra. The population dynamics obtained in the two different excitation conditions present striking differences due to inhomogeneous broadening. When the nanorings are excited at 720 nm, a non-single exponential relaxation in the excited state leads to a 10 nm redshift within the first picoseconds after photoexcitation, accompanied by the appearance of a more intense stimulated emission. These early-time dynamics are remarkably different to the data obtained upon excitation of the low-energy edge of the inhomogeneously broadened steady-state absorption at 860 nm. In that case the transient spectra present strong and prompt stimulated emission from a strongly allowed region of the S_1_ potential energy surface, whose amplitude decays by 20% within the first couple of ps after photoexcitation populating a region of the excited state potential with lower transition dipole moment. The differences between the spectra obtained in the two excitation conditions vanish for longer pump-probe delay times, suggesting the excited state structural dynamics are complete. Such differences in the fast dynamics of porphyrin nanorings are thus assigned to variation of transition dipole moments across the inhomogeneously broadened population. The observed dynamics are consistent with displaced minima and different curvatures for the ground and excited state potential energy surfaces along the torsional coordinate.

These data provide new information on the interplay between structural dynamics, disorder and spectroscopy in these nanoring systems. For example, we can conclude that static disorder, arising from a distribution of torsional angles between neighbouring chromophores, has a positive impact on the light harvesting capabilities of porphyrin nanorings. In *c*-P*n*_por_, the oscillator strength is effectively redistributed across a wider range of energies by the static disorder. Such inhomogeneously broadened spectra allow more efficient collection of red-NIR photons between 680 and 850 nm. The energy of these photons is then quickly (2–3 ps) funnelled to an excited state minimum, from where excitation energy transfer (EET) to a suitable acceptor can take place, thus potentially enhancing operation of photovoltaic devices. More generally, the present results show that static disorder can be exploited as a way of enhancing the spectral coverage (hence efficiency) of large π-electron molecular and supramolecular systems for light harvesting applications.

Coherent vibrational wavepacket dynamics were observed as modulations of the signal amplitude with an 85 fs period, displaying amplitude nodes (π phase-jumps) in spectral regions corresponding to the steady-state absorption or photoluminescence maxima. This permits assignment of such oscillations to wavepackets evolving on either the ground or excited electronic states. In the context of EET, if the donor–acceptor energy gap is resonant with a localised molecular vibration (Zn–N stretching) of the donor (*c*-P*n*_por_), such vibration could provide access to additional energy transfer pathways, thus improving overall efficiency of EET, as shown by Dean *et al.*^27^ and Arsenault *et al.*^71^ among others.^29,72^

Impulsive Raman spectra of the ground and the excited state of each nanoring were obtained. The impulsive ground and excited state resonance Raman spectra of the *c*-P*n*_por_ nanorings are very similar. This is in contrast to their radically different excitonic structures. This result allowed us to conclude that in this class of macromolecules the nuclear wavepackets are localised on the Zn porphyrin repeating units and are only weakly influenced by the degree of electronic delocalisation.

The largest contribution to both the ground and the excited state low-frequency resonance Raman spectra of *c*-P*n*_por_ is a 370 cm^−1^ Zn–N stretching mode, previously observed at slightly higher frequency in a Zn porphyrin parent monomer. The second most intense peak, observed at 428 cm^−1^ in the ground states, is assigned to a stretching and in-plane bending mode of the methinic and pyrrolic C–C bonds. We speculate that such a mode is redshifted by 26 cm^−1^ in the excited electronic states due to alterations in the bonding character of the B_1g_ molecular orbitals upon electronic excitation of tetrapyrroles belonging to the *D*_2h_ point group.

## Data availability

The data that support the findings of this contribution are available from the corresponding authors upon reasonable request.

## Author contributions

SRM and IAH coordinated the project, GB collected steady-state and time-resolved data, analysed data and wrote the first draft of the manuscript, MJ and HLA designed and synthesised the nanorings, PR performed DFT calculations, IAH conceived the experiments and analysed data. All authors contributed to the discussion of the data and revising and editing the manuscript.

## Conflicts of interest

The authors declare no competing financial interest.

## Supplementary Material

SC-013-D2SC01971J-s001
