# Supplementary material for: Population and coherence dynamics in large conjugated porphyrin nanorings
Source: Chem Sci. 2022 Jul 26;13(33):9624–36. doi: 10.1039/d2sc01971j (PMC9400675; doi:10.1039/d2sc01971j)
Supplement: SC-013-D2SC01971J-s001 [file SC-013-D2SC01971J-s001.pdf]

Electronic Supplementary Material (ESI) for Chemical Science.
This journal is © The Royal Society of Chemistry 2022

ELECTRONIC SUPPLEMENTARY INFORMATION

Population and coherence dynamics in Large, Fully Conjugated, Porphyrin Nanorings

Giovanni Bressan,[1] Michael Jirasek,[2] Palas Roy,[1] Harry L. Anderson,[2] Stephen R. Meech[1,*] and Ismael A. Heisler[3,*]

[1]*School of Chemistry, Norwich Research Park, University of East Anglia, Norwich NR4 7TJ, United Kingdom*

[2]*Department of Chemistry, University of Oxford, Chemistry Research Laboratory, Oxford OX1 3TA, United Kingdom*[3]

[3]*Instituto de Física, Universidade Federal do Rio Grande do Sul, Avenida Bento Gonçalves, 9500, CEP 91501-970, Porto Alegre, Brazil*

Corresponding authors: s.meech@uea.ac.uk ; ismael.heisler@ufrgs.br

TABLE OF CONTENTS:

Experimental

Fig. S1 experimental setup used for the fsTA/FCS measurements

Fig. S2 comparison of time traces of cP40 at 842 and 1100 nm after 720 nm or 860 nm centred excitation

Fig. S3 comparison of cP40 transient spectra at T = 100 fs, 4.5 ps, 500 ps of cP40 after 720 nm or 860 nm centred excitation

Fig. S4 probe wavelength-resolved GS and ES femtosecond coherence spectra of cP10-30

Fig. S5 Global fit residuals of cPnpor showing oscillatory behaviour on sub-ps timescale and time domain single damped sinusoid fit of c-P40 residuals (860 nm pump, 835 nm probe)

Fig. S6 probe wavelength-resolved FT amplitude and phase of the 370 cm^{-1} mode in the ground and excited electronic states of *c*-Pn_{por}

Fig. S7 probe wavelength-resolved impulsive Raman maps of *c*-P30 excited by 720 nm and 860 nm broadband pump pulses.

Fig. S8 probe wavelength-resolved impulsive Raman maps of *c*-P10, 20 and 40 excited by 720 nm and 860 nm broadband pump pulses.

Experimental

Butadiyne-bridged, Zn porphyrin nanorings, *c*-Pn_{por} (in which n_{por} is the number of constituent monomers and n_{por} = 10, 20, 30 or 40) have been synthesised as previously reported.[1–3] All samples were dissolved in toluene with 1% volume pyridine, to coordinate the Zn atoms and so avoid aggregation. The concentration of each sample has been adjusted in order to obtain a peak optical density of 0.3 in a 1 mm path cell.

The spectrometer is based on a design proposed by Riedle *et al.*[4] and has been described previously.[5] A diagram of the spectrometer is given in Fig. S1. Briefly, a titanium sapphire (Ti:Sa) chirped pulse amplifier (CPA), seeded by a Ti:Sa oscillator and pumped by a Q-switched Nd:YLF laser (Spectra-Physics) produces 5 mJ, 100 fs, 800 nm pulses at 1 kHz. 10% of the amplifier output is used to drive a noncollinear optical parametric amplifier (NOPA) and a separate collinear optical parametric amplifier (OPA) (Light Conversion). The NOPA produces broadband pulses, here centred at either 720 nm or 860 nm corresponding to high or low energy sides of the nanoring absorbance. These are used as pump pulses after recompression to ca. 25 fs by means of a folded two-prism compressor, and attenuation to a pulse energy of 10 nJ which avoids any effect of exciton-exciton annihilation.[6] The collinear OPA output is set at 1250 nm and used as a seed for generation of supercontinuum white light (SWL) in a 3 mm static sapphire window. The white light spectrum spans 550-1150 nm and is used as the probe.

The pump-probe delay time is scanned by means of a silver-coated retroreflector mounted on a linear translation stage (Physik Instrumente) in the pump path. The relative pump-probe polarisation is set at 45°, a cube beam splitter placed after the sample separates the parallel and perpendicular components of the transient molecular response, which are both dispersed by a home-built two-channel prism spectrometer and recorded on two identical 122x1024 pixels full-frame transfer CCD detectors (Stresing). The isotropic response (S_{iso}) is then retrieved as a combination of the signals measured on the two detectors, using:[7]

$$S_{iso} = \frac{(S_{\parallel} + 2S_{\perp})}{3} \qquad (1)$$

In which $S_{\parallel}$ and $S_{\perp}$ are the responses measured for relative parallel and perpendicular pump-probe polarisation conditions, respectively.

The pump-probe delay is scanned from -500 to +1000 fs in 8 fs steps, and then in longer time steps up to 3 ns, each reported data point is the result of the average of 500 pump on minus pump off difference spectra and furthermore, a spectrum at a negative delay time is subtracted from the whole dataset to correct for pump scatter and fluorescence contributions. Each probe wavelength-time delay surface is obtained as the average of 9 consecutive scans. Femtosecond coherence spectroscopy (FCS) data are obtained upon subtraction of the multiexponential global fits to the slowly decaying population components from each dataset. Truncation at 1 ps, followed by zero padding, numerical Fourier transformation of each probe wavelength with respect to the pump-probe delay time (T) and

integration over the relevant probe wavelengths allowed retrieval of impulsive resonant Raman spectra.[8]

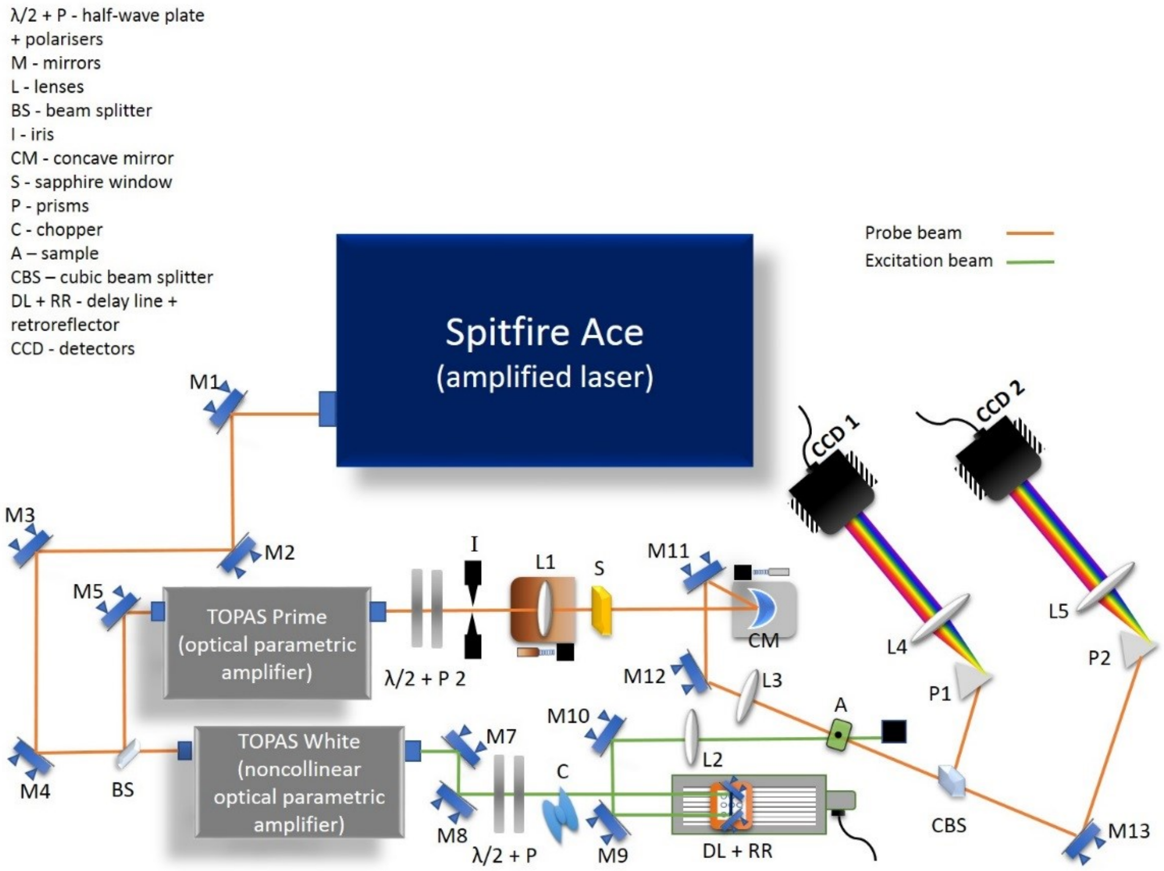

Fig. S 1 Sketch of the experimental setup used for the fsTA/FCS measurements shown in the main text, in which λ/2 + P are half-wave plates and polarisers, M are mirrors, L are lenses, I is an iris, BS is a beam splitter, CBS is a cubic beam splitter, CM is a concave mirror, S is a 3 mm thick sapphire window, P are prisms, C is a chopper, A is the sample, DL + RR are the delay line and retroreflector mounted on it and CCD 1/2 are the detectors measuring parallel and perpendicular response components, respectively. The CBS allows the parallel and perpendicular components of the signal to be separated.

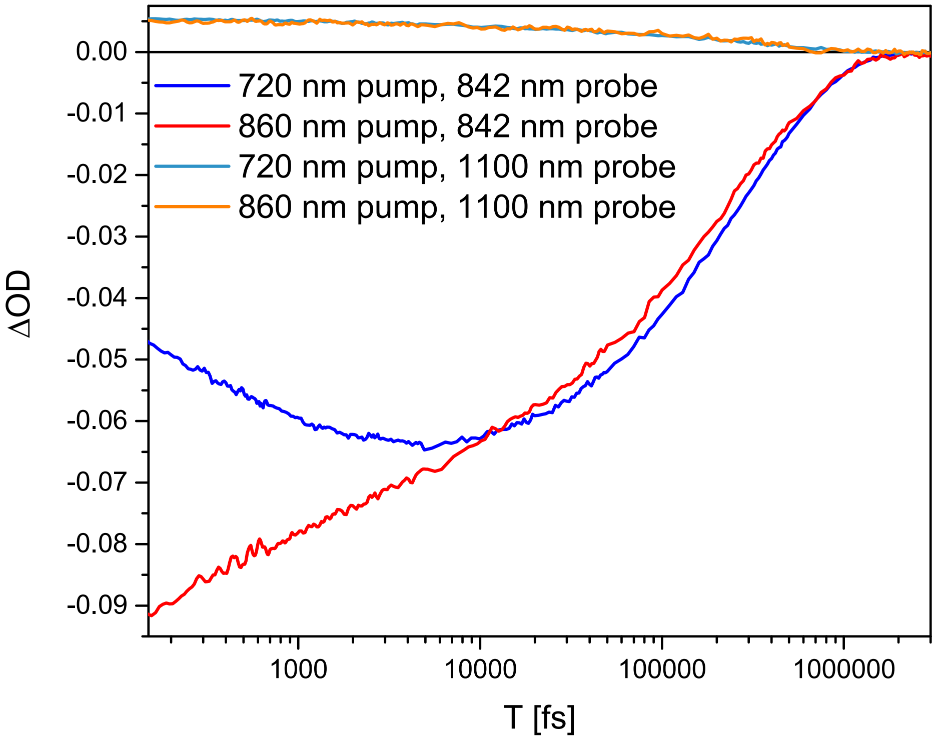

Figure S 2 fsTA time delay T vs difference OD traces at 842 nm after excitation with 720 nm (blue) and 860 nm (red) centred pump pulses for c-P40. T is reported on log10 scale to highlight the difference in early-time behaviour upon excitation with pump pulses centred at the different wavelengths.

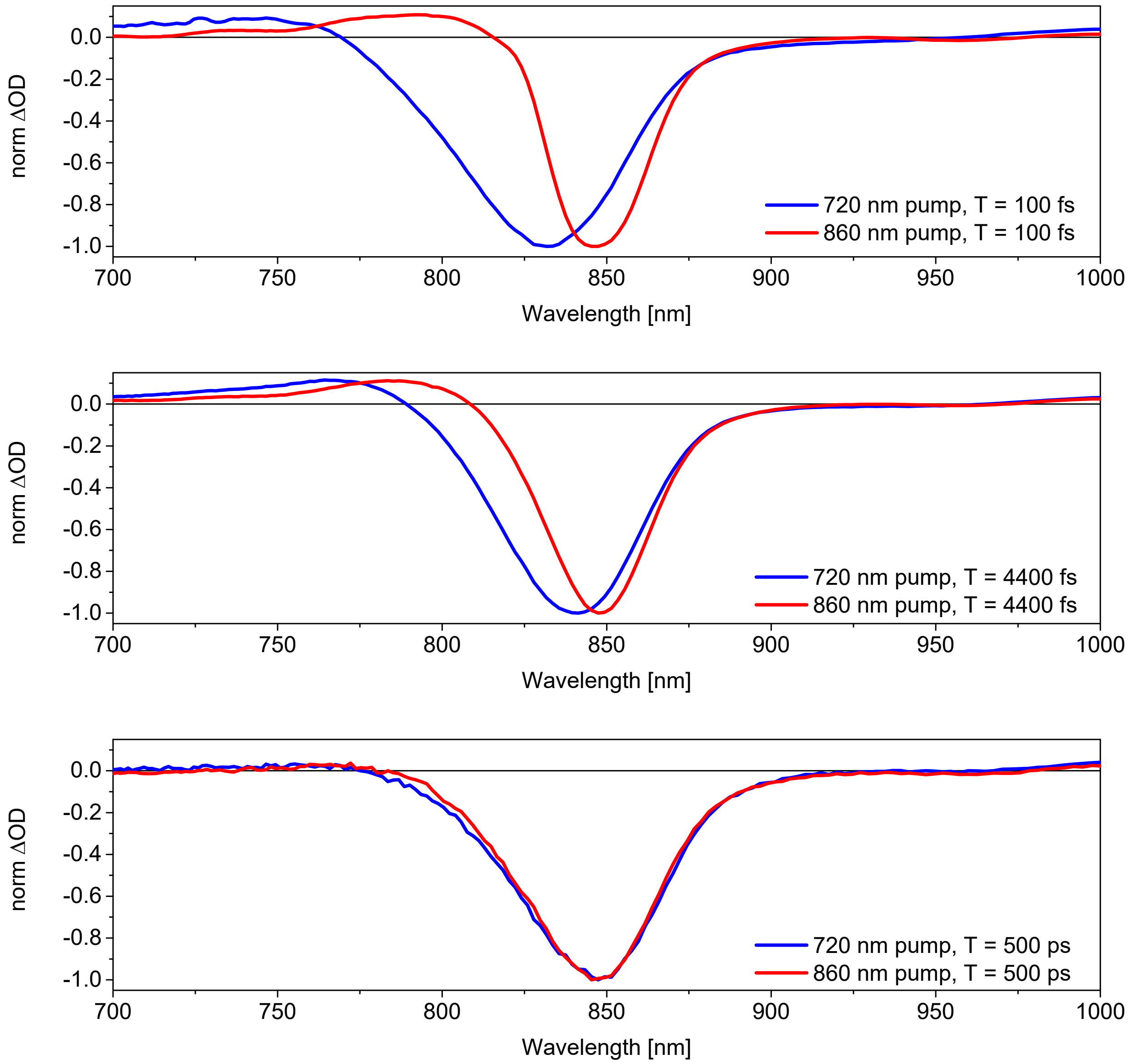

Figure S 3 Transient absorption spectra for c-P40 at two different excitation conditions, 720 nm (blue) and 860 nm (red) centred pump pulses. The different graphs correspond to different pump–probe delay times as for top T = 0.1 ps, middle T = 4.5 ps, and bottom T = 500 ps.

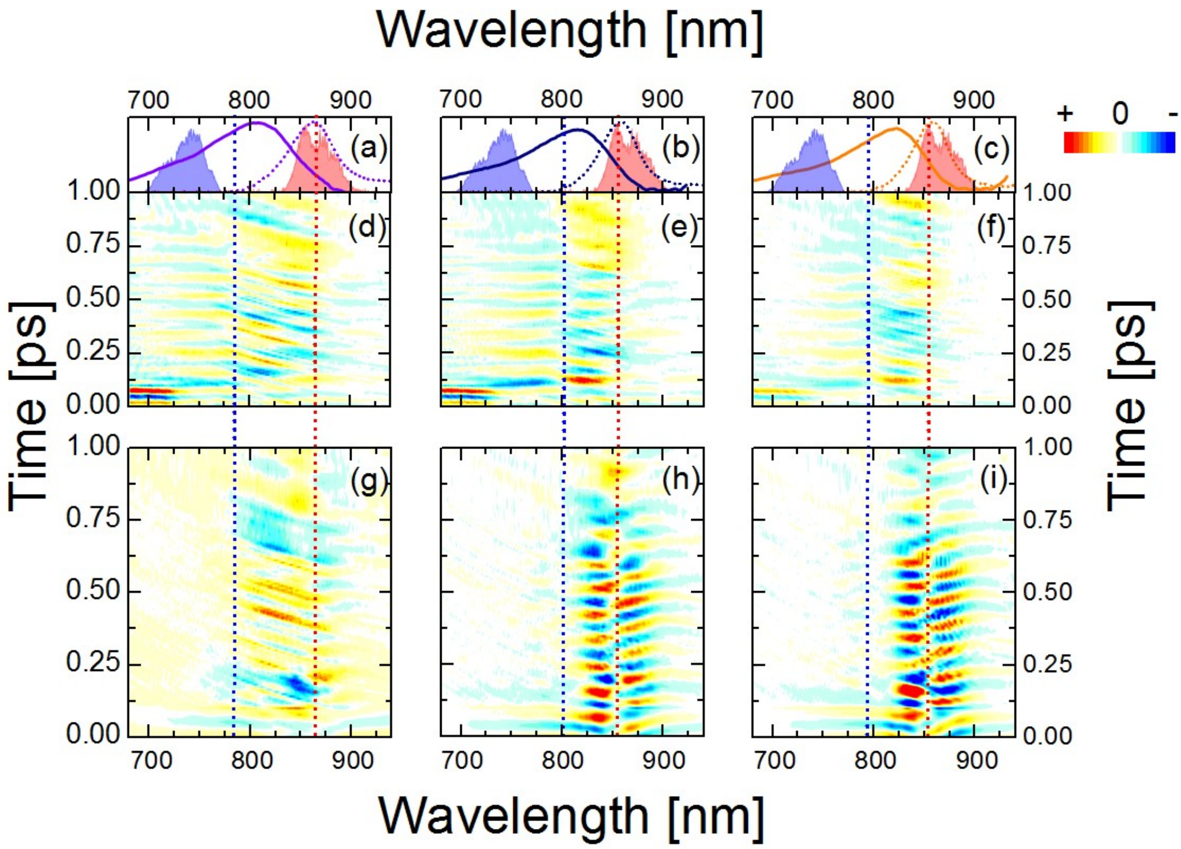

Fig. S 4 Femtosecond coherence spectra of c-P10 (d, g), c-P20 (e, h) and c-P30 (f, i) after excitation by 720 nm (d, e, f) or 860 nm (g, h, i) broadband pump pulses. Normalised steady-state absorption and PL spectra of c-P10 (a, violet), c-P20 (b, navy) and c-P30 (c, orange) are reported as solid and dashed lines, respectively, together with the normalised spectra of pump pulses centred at 720 nm and 860 nm (blue and red dashed areas in a-c). Vertical dashed lines highlight the spectral positions at which the phase-flips occur in both the ground (blue) and excited (red) potential energy surfaces of c-P10-30.

(a)

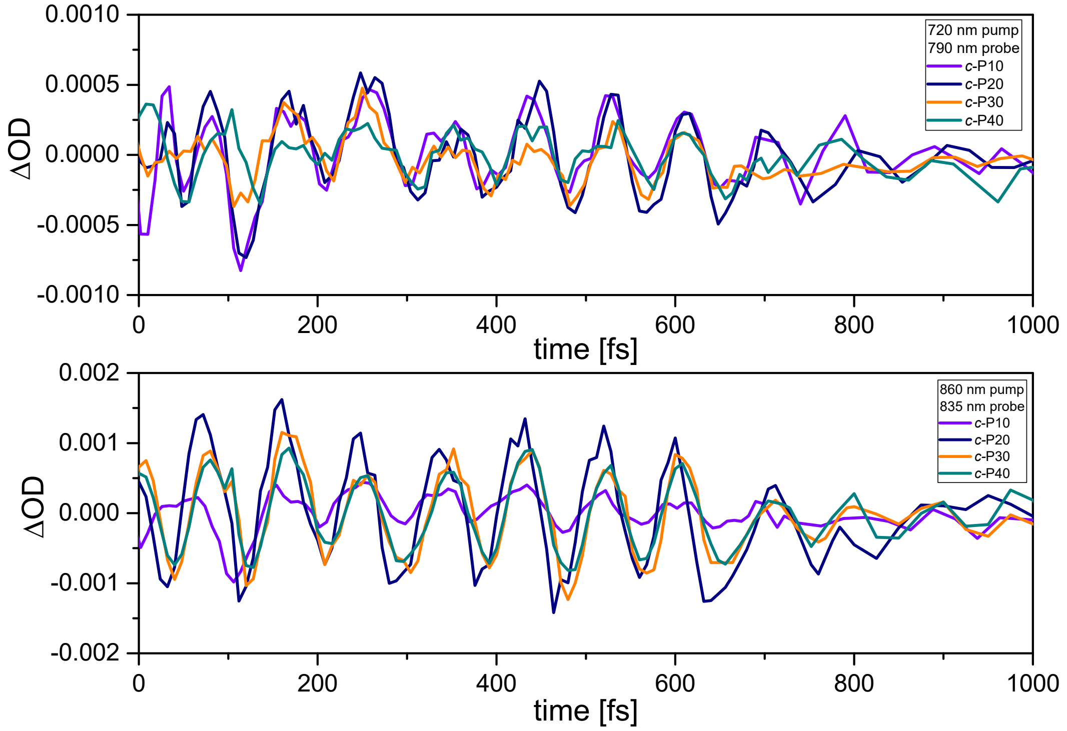

(b)

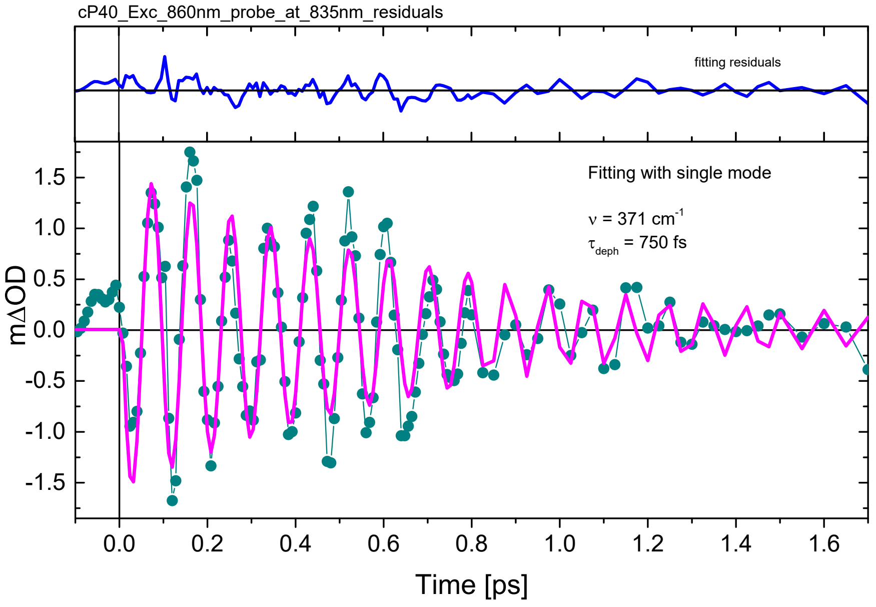

Fig. S 5 (a) Sub-ps oscillatory behaviour of the residuals of cPnpor after excitation at 720 (top) or 860 nm (bottom). (b) Time-domain fit of the residuals of c-P40 excited at 860 nm and probed at 835 nm. A single damped sinusoid with frequency of 371 cm^{-1} and dephasing time of 750 fs yields a good fit to the experimental data. The extracted parameters are in good agreement with the Fourier domain data.

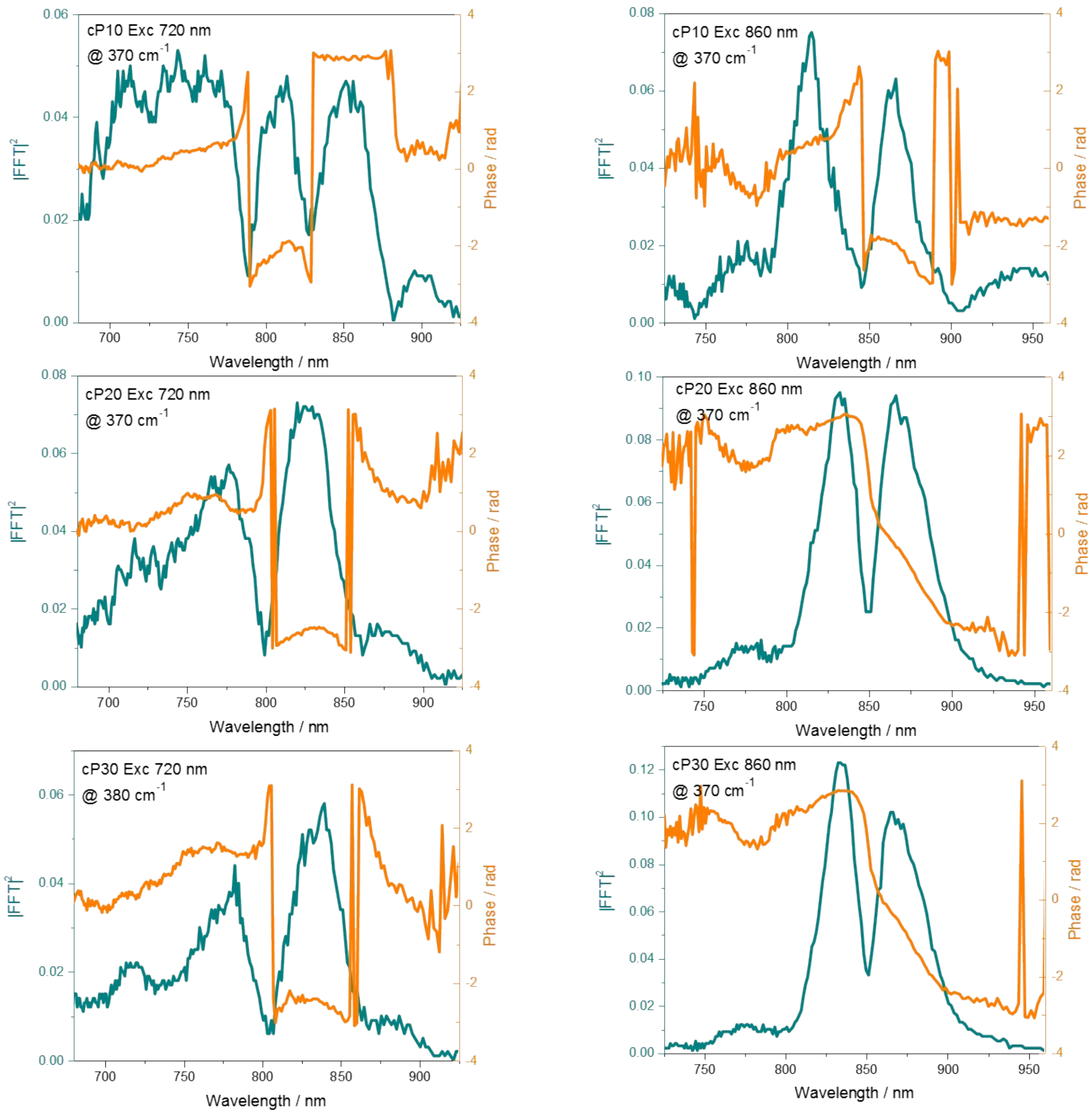

Fig. S 6 probe wavelength-resolved amplitude (teal solid line) and phase (orange solid line) of the 370 cm^{-1} mode in the ground (left column) and excited (right column) electronic states of c-P10 (top row), 20 (middle row) and 30 (bottom row).

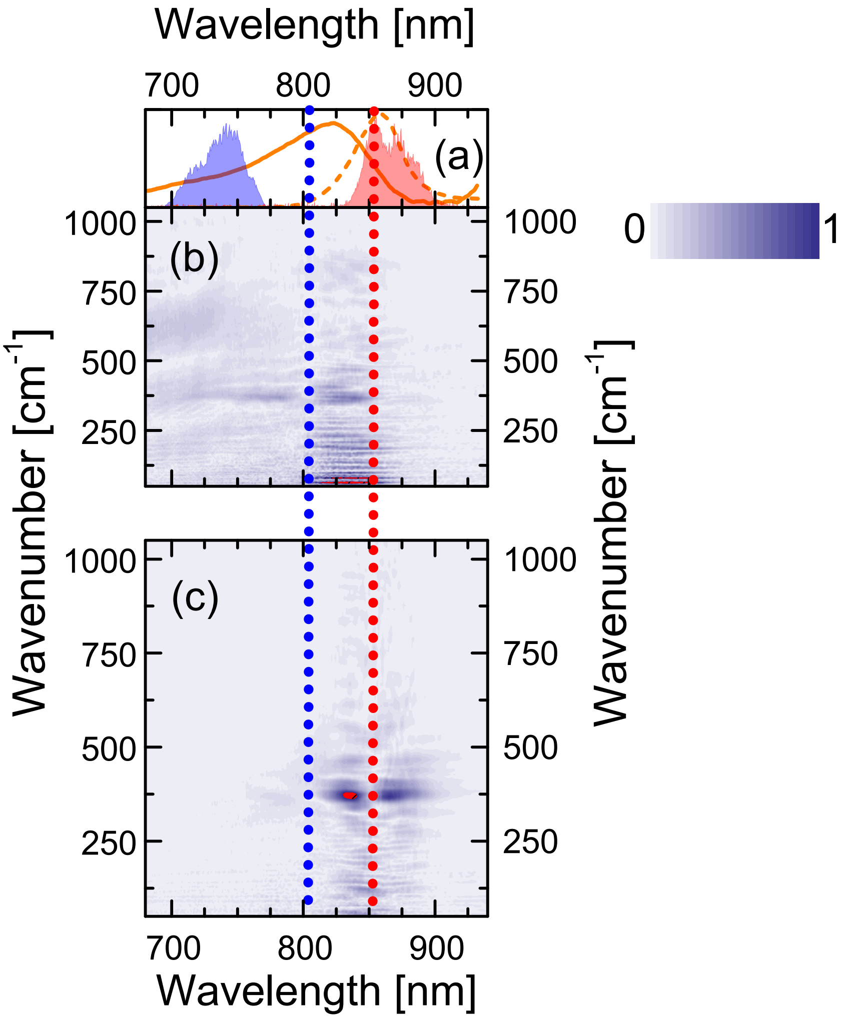

Fig. S 7 Probe wavelength-resolved impulsive Raman maps of c-P30 after impulsive excitation by 720 nm (b) or 860 nm (c) broadband pump pulses. Normalised steady-state absorption and PL spectra of c-P30 are reported as solid and dashed orange lines together with the normalised spectra of pump pulses centred at 720 nm and 860 nm (blue and red shaded areas in (a)). Vertical dotted lines highlight the spectral positions at which the phase-flips occur in both the ground (blue) and excited (red) potential energy surfaces of c-P30. Each Raman map is normalized to its maximum. The red dot shows saturation of an intense peak, required to improve visibility of weaker signals.

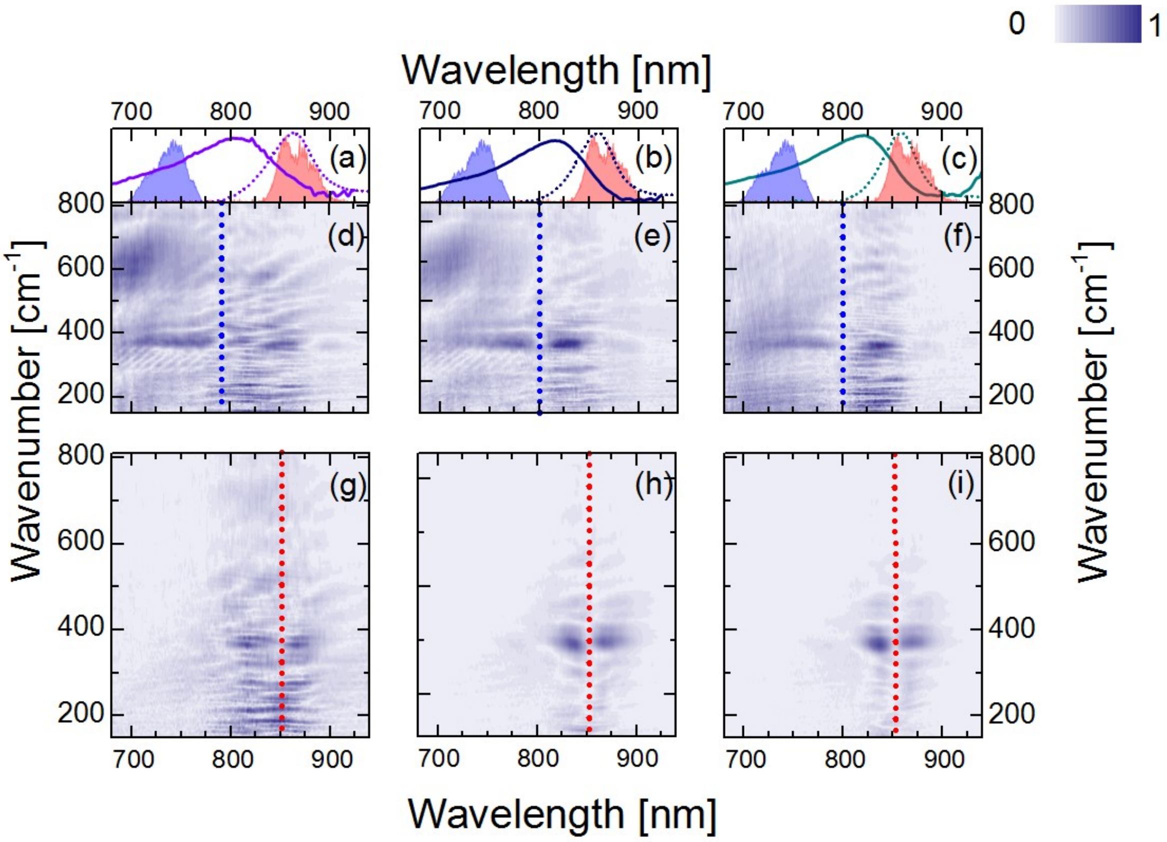

Fig. S 8 Probe wavelength-resolved impulsive Raman maps of c-P10 (d, g), c-P20 (e, h) and c-P40 (f, i) after impulsive excitation by 720 nm (d, e, f) or 860 nm (g, h, i) broadband pump pulses. Normalised steady-state absorption and PL spectra of c-P10 (a, violet), c-P20 (b, navy) and c-P40 (c, teal) are reported as solid and dashed lines together with the normalised spectra of pump pulses centred at 720 and 860 nm (blue and red shaded areas in a-c). Vertical dotted lines highlight the spectral positions at which the phase-flips occur in both the ground (blue) and excited (red) potential energy surfaces of c-P20-40. Each Raman map is normalized to its maximum.

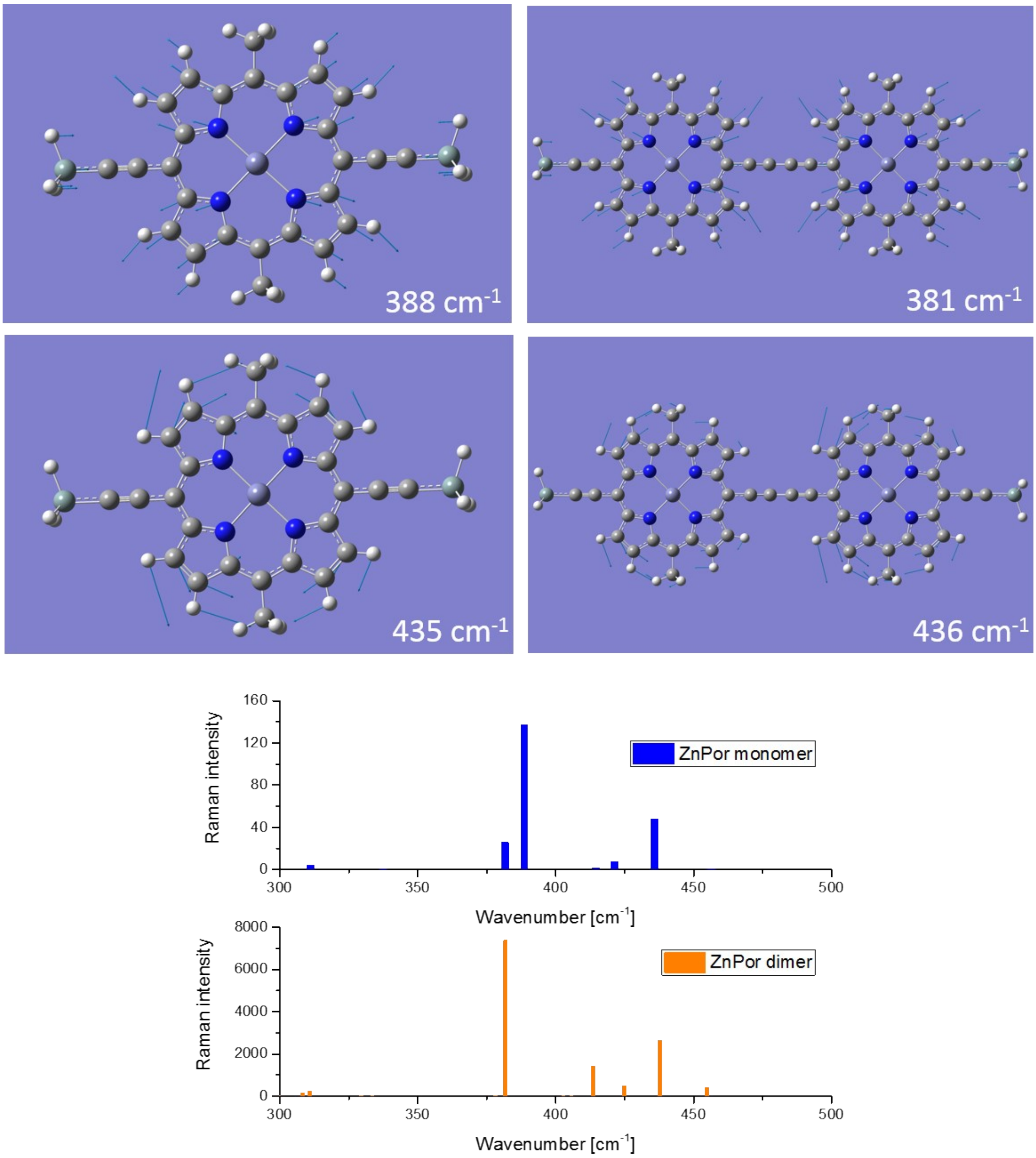

Fig. S 9 Sketches of the Zn-N stretching and of the methinic and pyrrolic in plane bending/stretching, and stick off-resonance GS Raman spectra of ZnPor monomer and ZnPor butadiyne linked dimer. These results were obtained by DFT, using Gaussian 16 at the B3LYP/6-31G level of theory.*[9]

Off-resonance ground state Raman spectra of the Zn porphyrin monomer and of the butadyine-linked dimer were calculated using Gaussian 16 at the B3LYP/6-31G* level of theory.[9] These calculations reproduce a redshift of the Zn-N stretching from the monomer (388 cm^{-1}) to the dimer (381 cm^{-1}), while the in-plane methinic/pyrrolic bending mode appears at essentially the same frequency (435 cm^{-1}) in monomer and dimer (436 cm^{-1}), in agreement with the experimental results.

Notes and References

[1] P.S. Bols and H.L. Anderson, Acc. Chem. Res. **51**, 2083 (2018).

[2] D. V. Kondratuk, L.M.A. Perdigao, M.C. O'Sullivan, S. Svatek, G. Smith, J.N. O'Shea, P.H. Beton, and H.L. Anderson, Angew. Chemie Int. Ed. **51**, 6696 (2012).

[3] P. Parkinson, D. V. Kondratuk, C. Menelaou, J.Q. Gong, H.L. Anderson, and L.M. Herz, J. Phys. Chem. Lett. **5**, 4356 (2014).

[4] U. Megerle, I. Pugliesi, C. Schriever, C.F. Sailer, and E. Riedle, Appl. Phys. B Lasers Opt. **96**, 215 (2009).

[5] G. Bressan, A.N. Cammidge, G.A. Jones, I.A. Heisler, D. Gonzalez-Lucas, S. Remiro-Buenamañana, and S.R. Meech, J. Phys. Chem. A **123**, 5724 (2019).

[6] G. Bressan, M. Jirasek, H.L. Anderson, I.A. Heisler, and S.R. Meech, J. Phys. Chem. C **124**, 18416 (2020).

[7] G. Bressan, M.D. Peeks, H.L. Anderson, S.R. Meech, and I.A. Heisler, J. Phys. Chem. C **123**, 27222 (2019).

[8] C. Fitzpatrick, J.H. Odhner, and R.J. Levis, J. Phys. Chem. A **124**, 6856 (2020).

[9] Gaussian 16, Revision C.01, M. J. Frisch, G. W. Trucks, H. B. Schlegel, G. E. Scuseria, M. A. Robb, J. R. Cheeseman, G. Scalmani, V. Barone, G. A. Petersson, H. Nakatsuji, X. Li, M. Caricato, A. V. Marenich, J. Bloino, B. G. Janesko, R. Gomperts, B. Mennucci, H. P. Hratchian, J. V. Ortiz, A. F. Izmaylov, J. L. Sonnenberg, D. Williams-Young, F. Ding, F. Lipparini, F. Egidi, J. Goings, B. Peng, A. Petrone, T. Henderson, D. Ranasinghe, V. G. Zakrzewski, J. Gao, N. Rega, G. Zheng, W. Liang, M. Hada, M. Ehara, K. Toyota, R. Fukuda, J. Hasegawa, M. Ishida, T. Nakajima, Y. Honda, O. Kitao, H. Nakai, T. Vreven, K. Throssell, J. A. Montgomery, Jr., J. E. Peralta, F. Ogliaro, M. J. Bearpark, J. J. Heyd, E. N. Brothers, K. N. Kudin, V. N. Staroverov, T. A. Keith, R. Kobayashi, J. Normand, K. Raghavachari, A. P. Rendell, J. C. Burant, S. S. Iyengar, J. Tomasi, M. Cossi, J. M. Millam, M. Klene, C. Adamo, R. Cammi, J. W. Ochterski, R. L. Martin, K. Morokuma, O. Farkas, J. B. Foresman, and D. J. Fox, Gaussian, Inc., Wallingford CT, 2016.
